# Human Parainfluenza Virus in Homeless Shelters before and during the COVID-19 Pandemic, Washington, USA

**DOI:** 10.3201/eid2811.221156

**Published:** 2022-11

**Authors:** Eric J. Chow, Amanda M. Casto, Reigran Sampoleo, Margaret G. Mills, Peter D. Han, Hong Xie, Brian Pfau, Tien V. Nguyen, Jaydee Sereewit, Julia H. Rogers, Sarah N. Cox, Melissa A. Rolfes, Constance Ogokeh, Emily Mosites, Timothy M. Uyeki, Alexander L. Greninger, James P. Hughes, M. Mia Shim, Nancy Sugg, Jeffrey S. Duchin, Lea M. Starita, Janet A. Englund, Pavitra Roychoudhury, Helen Y. Chu

**Affiliations:** University of Washington, Seattle, Washington, USA (E.J. Chow, A.M. Casto, R. Sampoleo, M.G. Mills, P.D. Han, H. Xie, B. Pfau, T.V. Nguyen, J. Sereewit, J.H. Rogers, S.N. Cox, A.L. Greninger, J.P. Hughes, M.M. Shim, N. Sugg, J.S. Duchin, L.M. Starita, P. Roychoudhury, H.Y. Chu);; Fred Hutchinson Cancer Research Center, Seattle (A.M. Casto, A.L. Greninger, J.P. Hughes, P. Roychoudhury);; Brotman Baty Institute for Precision Medicine, Seattle (P.D. Han, H. Xie, B. Pfau, L.M. Starita);; Centers for Disease Control and Prevention, Atlanta, Georgia, USA (M.A. Rolfes, C. Ogokeh, E. Mosites, T.M. Uyeki);; Military and Health Research Foundation, Laurel, Maryland, USA (C. Ogokeh);; Public Health Seattle and King County, Seattle (M.M. Shim, J.S. Duchin);; University of Washington Seattle Children’s Research Institute, Seattle (J.A. Englund)

**Keywords:** human parainfluenza virus, HPIV, viruses, epidemiology, homeless shelters, coronavirus disease, COVID-19, respiratory infections, zoonoses, King County, Washington, United States, severe acute respiratory syndrome coronavirus 2, SARS-CoV-2

## Abstract

To determine the epidemiology of human parainfluenza virus in homeless shelters during the COVID-19 pandemic, we analyzed data and sequences from respiratory specimens collected in 23 shelters in Washington, USA, during 2019–2021. Two clusters in children were genetically similar by shelter of origin. Shelter-specific interventions are needed to reduce these infections.

Human parainfluenza virus (HPIV) contributes to acute respiratory tract infection burden in young children (*1*) and adults (*2*). Persons experiencing homelessness are among those at risk for respiratory viral complications caused by chronic disease burden, mental illness, and social inequities. Homeless shelters might lack resources to reduce viral transmission by using nonpharmaceutical interventions (NPIs). We describe HPIV epidemiology in homeless shelters in King County, Washington, USA, before and during the COVID-19 pandemic.

We analyzed respiratory virus surveillance data from 2 previously described homeless shelter studies (*3*,*4*) conducted during October 2019‒May 2021. Eligible participants were residents at 1 of 23 homeless shelters who were >3 months of age and had a cough or >2 other acute respiratory illness symptoms. At enrollment, consenting participants or guardians completed questionnaires, and upper respiratory specimens were collected; each enrollment was considered 1 encounter. Once a month, persons were eligible to enroll, regardless of symptoms. Beginning April 1, 2020, enrollment expanded to residents and staff, regardless of symptoms. Participants could enroll multiple times; encounters were linked by name and birthdate.

We tested samples by using a TaqMan reverse transcription PCR platform that included influenza virus (A, B, C), respiratory syncytial virus, HPIV (*1*–*4*), human coronaviruses, rhinovirus, enterovirus, human bocavirus, human parechovirus, human metapneumovirus, adenovirus, and SARS-CoV-2 (beginning January 1, 2020). A cycle threshold value was generated. We typed HPIV-positive specimens, performed whole-genome sequencing by using hybrid capture on specimens that had a cycle threshold value <22 (Appendix), and submitted genomes to GenBank (Appendix Table 1). We aligned shelter consensus genomes generated with corresponding HPIV type genomes from GenBank, generated type-specific phylogenetic trees, and visualized trees by using NextStrain Auspice software (https://github.com). We analyzed the data descriptively by using SAS software version 9.4 (https://www.sas.com).

During October 2019‒May 2021, the study conducted 14,464 encounters with 3,281 unique participants (median age 37 years, range 0.3–85 years; 16% children; 17% shelter staff) (Appendix Figure 1). Among 1,569 encounters with positive virus test results, 32 (2%) encounters from 29 unique participants were HPIV positive (median age 29 years, range 0.3–64 years; 62% children, 45% female, 52% white, 100% resident; 10% had >1 chronic condition) (Appendix Table 2). Most HPIV-positive encounters (72%) occurred before April 1, 2020, and the highest HPIV-positive percentage was observed in family shelters (Table).

**Table:** Human parainfluenza virus detection across 23 homeless shelters, King County, Washington, USA, October 2019‒May 2021*

Time period	Type of shelter	Total	Human parainfluenza virus, no. (%) positive	Human parainfluenza virus types
Before April 1, 2020	Shelters: family (sites D, E, O)	303	16 (5.3)	HPIV-1, n = 5; HPIV-3, n = 6; HPIV-4, n = 5;
	Shelters: adults 18–25 y (site C)	89	1 (1.1)	HPIV-1, n = 1
	Shelters: adults >18 y (sites A, B, F, L)	845	3 (0.4)	HPIV-1, n = 2; HPIV untyped, n = 1
	Shelters: adults >50 y (site M)	453	3 (0.7)	HPIV-1, n = 2; HPIV untyped, n = 1
After April 1, 2020	Shelters: family (sites: D, E, H, N, O, OF, OG)	4,764	8 (0.2)	HPIV-3, n = 5; HPIV untyped, n = 3
	Shelters: adults 18–25 y (sites C, OH)	1,228	0	NA
	Shelters: adults >18 y (sites A, B, F, G, J, K, L, OB, OD)	6,078	1 (0.02)	HPIV untyped, n = 1
	Shelters: adults >50 y (sites I, M, OA, OC, OE)	661	0	NA
Total		14,421†	32 (0.2)	HPIV-1, n = 10; HPIV-3, n = 11; HPIV-4, n = 5; HPIV untyped, n = 6

*A Washington State Stay-At-Home ordinance we issued on March 23, 2020. HPIV, human parainfluenza virus; NA, not available. †n = 43 encounters for which dates were missing were not included (none involved human parainfluenza-positive specimens).

Six of 32 encounters involved viral co-infections with HPIV (rhinovirus, adenovirus, human bocavirus, enterovirus, and human parechovirus). Participants with HPIV infection reported symptoms at 25 (78%) encounters. Commonly reported symptoms included rhinorrhea (95%), cough (74%), sore throat (53%), and subjective fevers (47%) (Appendix Table 3). HPIV-positive specimens occurred every month during October 2019‒April 2020 (Appendix Figure 2). Only 2 HPIV infections were identified during May 2020‒April 2021, despite an average of 954 monthly encounters. Six HPIV infections occurred during May 2021 (Appendix Figure 3).

Of 32 HPIV-positive specimens, we identified 3 of the 4 HPIV serotypes: 10 HPIV-1, 11 HPIV-3, and 5 HPIV-4. Six specimens were untypeable. Sequencing of 16 specimens generated 11 sequences (4 HPIV-1, 4 HPIV-3, and 3 HPIV-4a) from 6 shelters (Figure). HPIV-1 sequences formed 2 clusters (100% bootstrap support for each cluster) by collection date in a maximum-likelihood tree that included 94 GenBank HPIV-1 genomes. Both HPIV-3 and HPIV-4a sequences formed single genetic clusters (100% bootstrap support for each cluster) in a maximum-likelihood tree that included 397 GenBank HPIV-3 and 24 HPIV-4a genomes. The HPIV-3 clusters involved HPIV-positive specimens from shelters E (October 2019) and H (May 2021); both shelters housed adults and children. In shelter E, HPIV-3‒positive specimens resulted from 6 encounters involving 5 unique participants (all children) spanning 9 days, and 2 specimens were sequenced. In shelter H, 5 HPIV-3 encounters involving 4 unique participants (all children) spanned 17 days, and 2 specimens were sequenced. The sequenced HPIV-3 specimens from shelters E and H, each from unique persons, formed 2 subclusters, each with 100% bootstrap support, corresponding to shelter and collection date.

**Figure:**
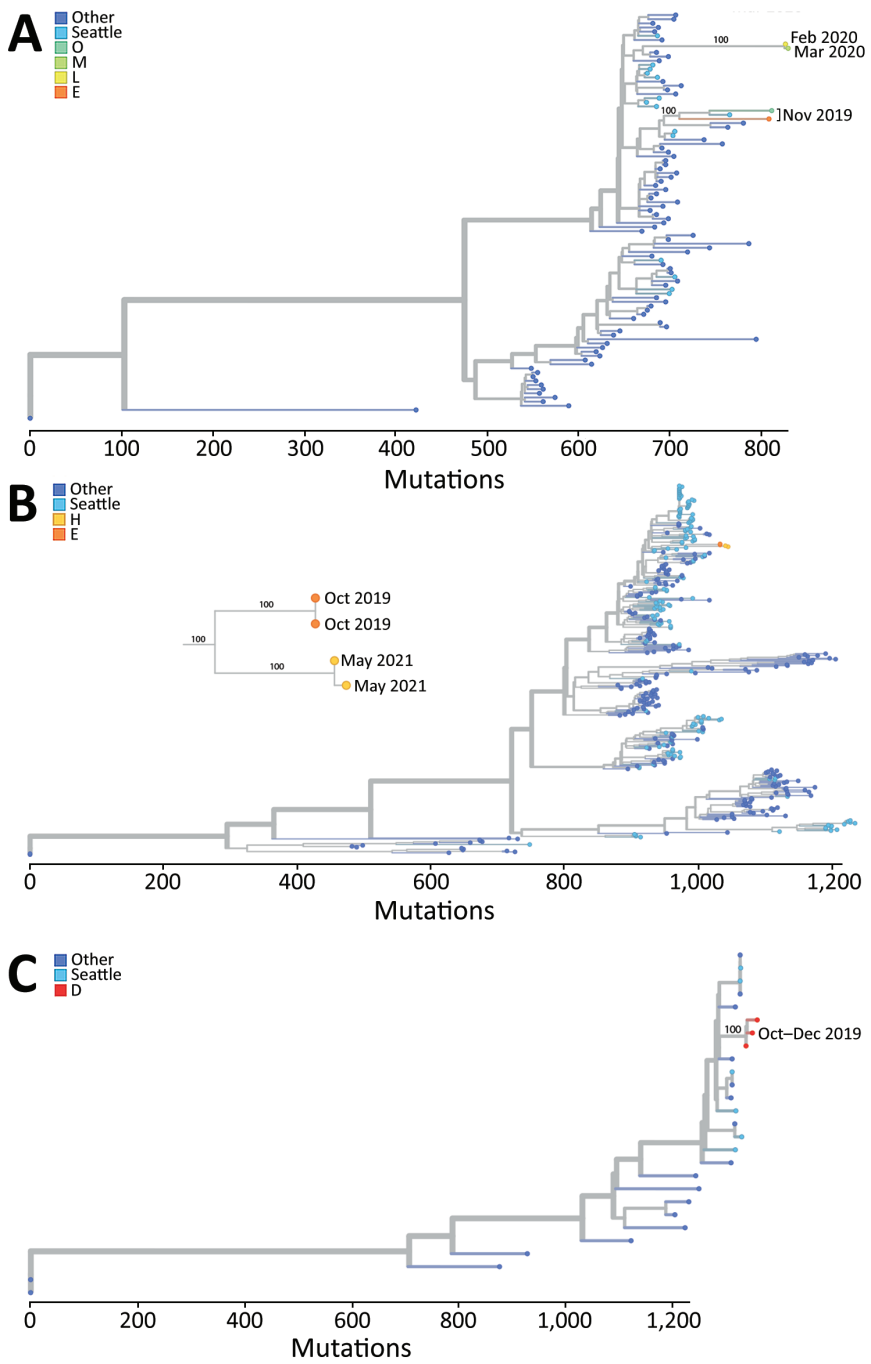
Phylogenetic trees of human parainfluenza viruses in homeless shelters, King County, Washington, USA, October 2019‒May 2021. A) Human parainfluenza virus 1; B) human parainfluenza virus 3; C) human parainfluenza virus 4a. Letters in keys indicate different homeless shelters from which sequenced specimens were collected. Other indicates genomic data from locations not in Seattle, Washington. Seattle indicates genomic data from Seattle other than homeless shelters in this study.

Respiratory viruses are increasingly appreciated as major pathogens in homeless shelters (*5*,*6*), We identified HPIV infections in shelter residents of all ages, although predominantly in children. Family shelters that have mixed populations of adults and children had the greatest percentage of HPIV detections. Two pediatric HPIV-3 clusters occurred before and during the COVID-19 pandemic with genetic clustering by shelter. After the Washington stay-at-home ordinance on March 23, 2020, overall numbers of HPIV infections decreased. These reductions (*7*) were probably in part caused by community implementation of NPIs because respiratory droplets are probably the main mode of HPIV transmission (*8*). However, HPIV has been detected on environmental surfaces (*9*), and shelter site resources might not enable adequate social distancing and air quality.

The pediatric HPIV-3 cases illustrate the need for mitigation guidance to reduce intrashelter HPIV transmission, particularly because younger children have higher upper respiratory tract viral levels than older persons (*10*). Limitations of this study included potential selection bias, a lack of site-specific NPI data, cross-sectional study design, and inability to compare concurrent shelter results to community HPIV epidemiology. These HPIV data provide information on site-specific characteristics to inform public health guidance.

Appendix Additional information on human parainfluenza virus in homeless shelters before and during the COVID-19 pandemic, Washington, USA.
