# Supplementary material for: Human Parainfluenza Virus in Homeless Shelters before and during the COVID-19 Pandemic, Washington, USA
Source: Emerg Infect Dis. 2022 Nov;28(11):2343–7. doi: 10.3201/eid2811.221156 (PMC9622239; doi:10.3201/eid2811.221156)
Supplement: Appendix — Additional information on human parainfluenza virus in homeless shelters before and during the COVID-19 pandemic, Washington, USA. [file 22-1156-Techapp-s1.pdf]

Article DOI: https://doi.org/10.3201/eid2811.221156

Human Parainfluenza Virus in Homeless Shelters before and during the COVID-19 Pandemic, Washington, USA

Appendix.

Supplemental Methods

We analyzed data from 2 previously described studies: 1) a randomized control trial of an influenza test and treat (NCT04141917) (*1*) and a SARS-CoV-2 surveillance study (*2*) of 23 homeless shelters across King County, Washington during October 2019–May 2021. These studies took place sequentially and data from cross-sectional survey responses and respiratory specimens were collected at study enrollment. Before April 1, 2020, eligible participants were shelter residents ≥3 months of age who had a cough or at least 2 other acute respiratory illness symptoms, including subjective fever, headache, sore throat, rhinorrhea, shortness of breath, and myalgias, and were recruited from shelter site staffed kiosks. For participants <18 years of age, diarrhea, rash, and ear pain or discharge were also included. Monthly enrollment of participants not meeting the symptom requirements were also permitted to enroll in the study for shelter surveillance. From April 1, 2020, onward, enrollment eligibility included residents and staff regardless of symptoms when SARS-CoV-2 community circulation was detected. Participants were not followed-up longitudinally, but were permitted to enroll multiple times. Multiple participant encounters were linked by name and birthdate. One-day surge testing events took place as part of Public Health Seattle and King County SARS-CoV-2 contact tracing efforts starting on March 30, 2020, in which study participation was offered to all residents and shelter staff with or without symptoms.

Study consent was obtained from all persons ≥18 years of age or from a guardian for persons <18 years of age. Study participant assent was required for those persons 13–17 years of age. After consent was obtained, an enrollment questionnaire was administered collecting information sociodemographics, current tobacco use, self-reported chronic conditions, and illness

course symptoms. Loss of taste or smell was included after April 1, 2020. Collected sociodemographic data included shelter site location, birthdate, sex at birth, race and ethnicity, pregnancy status, and current tobacco use status. Self-reported chronic conditions included neurologic disease, cardiovascular disease, asthma, bronchitis, chronic obstructive pulmonary disease, hepatic disease, diabetes mellitus, immunosuppression, and cancer or another condition not listed. Illness course questions in the survey included self-reported symptoms and illness duration. Questionnaire symptoms included rhinorrhea, cough, sore throat, fatigue, myalgias, headaches, subjective fevers, shortness of breath, sweats, nausea or vomiting, chills, diarrhea, rash, ear pain or discharge and loss of taste or smell (added after April 1, 2020). Participants not reporting any of these symptoms were considered asymptomatic up until study enrollment. Shelter facility specifics including targeted resident demographics that the shelter served were obtained from shelter management. Survey questionnaires were administered on an electronic tablet at the time of respiratory sample collection and data was stored on Research Electronic Data Capture. A respiratory sample was also collected at enrollment by using midturbinate sterile nylon flocked swabs (Copan Diagnostics, https://www.copanusa.com) with anterior nares swabs briefly used during July 22, 2020–November 1, 2020, because of COVID-19 pandemic–associated supply changes. Respiratory specimens were initially obtained by study staff. However, procedure was converted to study staff supervised self-collected swab specimens with the community spread of SARS-CoV-2. We prepared this analysis by using deidentified study data. The University of Washington IRB (study no. 00007800) approved this study.

Specimens were stored at 4°C in universal transport medium. Respiratory specimens were tested by using the TaqMan RT-PCR platform (Thermo Fisher OpenArray, https://www.thermofisher.com) that included influenza virus (A, B and C), respiratory syncytial virus (RSV-A and RSV-B), human parainfluenza (HPIV 1-4), human coronaviruses (HCoV-NL63, HCoV-OC43, HCoV-229E, HCoV-HKU1), rhinovirus, enterovirus, human bocavirus, human parechovirus, human metapneumovirus, adenovirus and SARS-CoV-2 (from January 1, 2020 onward). Specimens collected from January 1, 2020, onward were tested for SARS-CoV-2 with those collected after February 25, 2020 tested prospectively. For the purposes of this study, we categorized SARS-CoV-2 inconclusive results as negative. HPIV co-infections were defined as detection of HPIV and ≥1 other respiratory virus. A cycle threshold (Ct) was generated for each virus-positive sample.

Human Parainfluenza Typing

RNA was extracted from specimens by using the MagnaPure 96 DNA and Viral NA Small Volume Kit, (Roche, https://www.roche.com). Viral NA Universal SV 4.0 protocol (200 µL input, 50 µL elution) and were used for HPIV typing and sequencing. All 32 HPIV-positive specimens were typed by using a multiplex RT-PCR for HPIV-1, -3, and -4 (*3*) and a separate PCR for HPIV-2 (*4*).

Amplification reactions of 35-µL reactions contained 10 µL of extracted RNA and the AgPath-ID One Step RT-PCR enzyme and master mix (Ambion, https://www.thermofisher.com). Multiplex reactions contained 250 nmol/L each of forward and reverse primers for HPIV-1 and HPIV-3, 375 nmol/L each of forward and reverse primers for HPIV-4, and 100 nmol/L each of probes for HPIV-1, HPIV-3, and HPIV-4. HPIV-2 reactions contained 250 nmol/L each of forward and reverse primers and 100 nmol/L of probe. Amplification was conducted in ABI 7500 Thermocyclers (https://www.thermofisher.com) by using 10 minutes of reverse transcription at 48°C, 10 minutes of denaturation at 98°C, and 40 cycles of 15 seconds at 95°C and 45 seconds at 60°C. Specimens were considered positive if the PCR Ct value was <40 based on established cutoffs for laboratory-developed tests. HPIV-positive specimens that were not positive for HPIV 1-4 are listed as untyped.

Genomic Sequencing

HPIV sequencing was attempted on specimens that had with Ct values <22 by using an oligonucleotide probe capture panel targeting common respiratory viruses including HPIV. Sequencing libraries were prepared from extracted RNA by using the Respiratory Virus Oligo Panel version 2 (Illumina, https://www.illumina.com) according to the manufacturer's instructions. In brief, RNA was converted to double-stranded complementary DNA, and precapture libraries were prepared and normalized by using the Illumina RNA Prep with Enrichment (L) Tagmentation Kit. After hybridization with capture probes, the resulting enriched library was cleaned by using AMPure XP beads (https://www.beckmancoulter.com). Libraries were quantified by using the Quant-IT HS dsDNA Kit (https://www.thermofisher.com) on the Victor Nivo Multimode PlateReader https://www.thermofisher.com), and library size was checked by using the Agilent 4200 TapeStation system (https://www.agilent.com).

Human Parainfluenza Pipeline Description

Consensus genomes were generated by using a custom bioinformatic pipeline (https://github.com/greninger-lab/revica). In brief, raw reads were trimmed by using Trimmomatic version 0.39 (https://bioweb.pasteur.fr) and the settings ILLUMINACLIP:2:30:10:1:true, SLIDINGWINDOW: 4:20, LEADING: 3, TRAILING: 3, and MINLEN: 35. Trimmed reads were mapped to a multi-fasta reference containing complete genomes of human parainfluenza virus by using BBMap version 38.96 (https://anaconda.org). The reference with the highest median coverage was selected as the initial reference for consensus calling. Trimmed reads were then mapped again to the initial reference by using BBMap, and the resulting alignment was used to call a consensus genome by using Samtools version 1.15 (http://www.htslib.org) and iVar version 1.3.1 (https://github.com). A minimum coverage of 3, a minimum base quality of 15, and a minimum frequency threshold of 0.6 were required to call consensus. Regions with less than the minimum coverage were called Ns. This process of reference-based consensus calling was iterated 3 times, and leading and trailing Ns were trimmed to generate a final consensus. Generated consensus genomes were submitted to GenBank under accessions nos. ON778017–ON778027 (Appendix Table 1).

Computational Analysis

Publicly available parainfluenza genome sequences were downloaded from GenBank. Consensus genomes generated for this project were aligned with others of the same subtype from GenBank by using MAFFT version 7.453 (https://mafft.cbrc.jp). Maximum-likelihood phylogenetic trees were generated for each subtype, and bootstrap values were calculated by using IQ-Tree version 2.2.0 (www.iqtree.org). Trees were visualized by using NextStrain Auspice software (https://docs.nextstrain.org). We analyzed our data descriptively by using SAS software version 9.4 (https://www.sas.com).

References

1. Newman KL, Rogers JH, McCulloch D, Wilcox N, Englund JA, Boeckh M, et al.; Seattle Flu Study Investigators. Point-of-care molecular testing and antiviral treatment of influenza in residents of homeless shelters in Seattle, WA: study protocol for a stepped-wedge cluster-randomized controlled trial. Trials. 2020;21:956. PubMed https://doi.org/10.1186/s13063-020-04871-5

2. Rogers JH, Link AC, McCulloch D, Brandstetter E, Newman KL, Jackson ML, et al.; Seattle Flu Study Investigators. Characteristics of COVID-19 in homeless shelters: a community-based surveillance study. Ann Intern Med. 2021;174:42–9. PubMed https://doi.org/10.7326/M20-3799

3. Fairchok MP, Martin ET, Kuypers J, Englund JA. A prospective study of parainfluenza virus type 4 infections in children attending daycare. Pediatr Infect Dis J. 2011;30:714–6. PubMed https://doi.org/10.1097/INF.0b013e3182113989

4. Kuypers J, Wright N, Ferrenberg J, Huang M-L, Cent A, Corey L, et al. Comparison of real-time PCR assays with fluorescent-antibody assays for diagnosis of respiratory virus infections in children. J Clin Microbiol. 2006;44:2382–8. PubMed https://doi.org/10.1128/JCM.00216-06

Appendix Table 1. GenBank accession numbers for human parainfluenza virus sequences

Sequence name	Collection date	Virus type	Shelter of origin	Participant age, years	GenBank accession no.
PIV1_1	2019 Nov 4	1	O	31	ON778017
PIV1_2	2019 Nov 22	1	E	1	ON778018
PIV1_3	2020 Feb 28	1	L	46	ON778019
PIV1_4	2020 Mar 31	1	M	64	ON778020
PIV3_1	2019 Oct 15	3	E	1	ON778021
PIV3_2	2019 Oct 21	3	E	3	ON778022
PIV3_3	2021 May 13	3	H	3	ON778023
PIV3_4	2021 May 25	3	H	4	ON778024
PIV4a_1	2019 Oct 28	4	D	11	ON778025
PIV4a_2	2019 Nov 14	4	D	2	ON778026
PIV4a_3	2019 Dec 3	4	D	0	ON778027

Appendix Table 2. Demographics and health characteristics of study participants*

Characteristic.	Human parainfluenza virus†	Other respiratory viruses‡	No respiratory virus detected§
No. unique participants	29	973	2,996
Age, years			
Overall, median (range)	20 (0.3–64)	33 (0.3–85)	37 (0.3–85)
<5	12 (44.4)	99 (10.2)	154 (5.1)
5–11	3 (11.1)	93 (9.6)	189 (6.3)
12–17	1 (3.7)	43 (4.4)	101 (3.4)
18–49	7 (25.9)	485 (49.9)	1,635 (54.6)
50–64	4 (14.8)	212 (21.8)	752 (25.1)
≥65	0	41 (4.2)	164 (5.5)
Sex			
M	15 (51.7)	570 (58.6)	1,815 (60.6)
F	13 (44.8)	389 (40.0)	1,127 (37.6)
Other	0	2 (0.2)	16 (0.5)
Prefer not to say	1 (3.5)	12 (1.2)	38 (1.3)
Race			
White	15 (51.7)	398 (40.9)	1,208 (40.3)
Black or African American	8 (27.6)	321 (33.0)	950 (31.7)
Asian	0	17 (1.8)	114 (3.8)
American Indian or Alaskan Native	1 (3.5)	30 (3.1)	121 (4.0)
Native Hawaiian or Pacific Islander	1 (3.5)	64 (6.6)	129 (4.3)
Other	1 (3.5)	64 (6.6)	263 (8.8)
Prefer not to say	3 (10.3)	79 (8.1)	211 (7.0)
Ethnicity			
Hispanic	3 (10.3)	155 (15.9)	440 (14.7)
Non-Hispanic	25 (86.2)	797 (81.9)	2,502 (83.5)
Unknown	1 (3.5)	21 (2.2)	54 (1.8)
Pregnancy status for women of child-bearing age	n = 5	n = 258	n = 770

Characteristic.	Human parainfluenza virus†	Other respiratory viruses‡	No respiratory virus detected§
Pregnant	1 (20.0)	5 (1.9)	13 (1.7)
Not pregnant	2 (40.0)	75 (29.1)	128 (16.6)
Prefer not to say	2 (40.0)	178 (69.0)	629 (81.7)
Smoking status			
Current tobacco use	6 (20.7)	405 (41.6)	1368 (45.7)
Chronic conditions			
None	26 (89.7)	699 (71.8)	2081 (69.5)
>1 chronic condition	3 (10.3)	274 (28.2)	915 (30.5)
Neurologic disease	0	23 (2.8)	63 (2.6)
Cardiovascular disease	0	23 (2.4)	95 (3.2)
Asthma	1 (3.5)	117 (12.0)	393 (13.1)
Bronchitis	1 (3.5)	24 (2.5)	93 (3.1)
Chronic obstructive pulmonary disease	1 (3.5)	37 (3.8)	116 (3.9)
Hepatic disease	1 (3.5)	21 (2.2)	85 (2.8)
Diabetes mellitus	1 (3.5)	62 (6.4)	199 (6.6)
Immunosuppression	0	14 (1.4)	36 (1.2)
Cancer	1 (3.5)	16 (1.6)	57 (1.9)
Other	0	11 (1.1)	31 (1.0)
Shelter staff	0	109 (11.2)	550 (18.4)
No. encounters	32	1,537	12,895

*Values are no. (%) where indicated. Participant groups were not mutually exclusive because some participants might have encounters for which human parainfluenza was detected and others for which other virus or no virus was detected.
†n = 2 encounters for which participant age is missing and were not included in the age analysis.
‡There were n = 22 encounters for which an inconclusive test result for severe acute respiratory syndrome virus 2 was recategorized as a negative result; there were no other pathogens detected in these specimens and 17 of these specimens came from asymptomatic participants.
§n = 1 encounter for which participant age is missing and was not included in the age analysis.

Appendix Table 3. Participant symptoms among those with human parainfluenza only*

Symptom	Human parainfluenza only	No virus detected
Encounters	26	12,895
Unique participants	25	2,996
Encounter symptoms, no. (%)		
Asymptomatic	7 (26.9)	10,700 (83.0)
Symptomatic	19 (73.1)	2,195 (17.0)
Rhinorrhea	18 (94.7)	1,277 (58.2)
Cough	14 (73.7)	1,070 (48.8)
Sore throat	10 (52.6)	662 (30.2)
Subjective fevers	9 (47.4)	459 (20.9)
Fatigue	8 (42.1)	749 (34.1)
Myalgias	6 (31.6)	711 (32.4)
Chills	6 (31.6)	444 (20.2)
Diarrhea	6 (31.6)	296 (13.5)
Shortness of breath	5 (26.3)	348 (15.9)
Sweats	4 (21.1)	427 (19.5)
Headaches	3 (15.8)	711 (32.4)
Ear pain or discharge	3 (15.8)	148 (6.7)
Rash	3 (15.8)	125 (5.7)
Nausea or vomiting	2 (10.5)	497 (22.6)
	n = 8	n = 1,904
Loss of taste or smell†	0	34 (1.6)
Influenza-like illness‡	9 (47.4)	353 (16.1)
COVID-19-like illness§	9 (47.4)	328 (14.9)
Influenza-like illness and COVID-19-like illness symptoms	9 (47.4)	315 (14.4)

*COVID-19, coronavirus disease.
†Loss of taste or smell was collected from April 1, 2020, onward.
‡Defined as fever with cough or sore throat.
§Defined as fever with cough or shortness of breath.

Appendix Table 4. Sequences of primers and probes used in this study*

HPIV type	Primer/probe name	Primer/probe sequence, 5′→3′
HPIV-1	Forward primer 1	CGA GCG AGT CGA TTT ATT ACC A
	Reverse primer 1	CAA TCC GGT TAA CAT AAT TTG A
	Probe 1	Cy5-TGG CAT TAA AAG AG+G C+AG +GA-BHQ
HPIV-2	Forward primer 2	TGC ATG TTT TAT AAC TAC TGA TCT TGC TAA
	Reverse primer 2	GTT CGA GCA AAA TGG ATT ATG GT
	Probe 2	VIC-ACT GTC TTC AAT GGA GAT AT-MGB
HPIV-3	Forward primer 3	TGC TGT TCG ATG CCA ACA A
	Reverse primer 3	ATT TTA TGC TCC TAT CTA GTG GAA GAC A
	Probe 3	FAM-TTG CTC TTG CTC A-MGB
HPIV-4	Forward primer 4	TGC CAA ATC GGC AAT TAA ACA
	Reverse primer 4	GGC TCT GGC AGC AAT CAT AAG
	Probe 4	VIC-TGA TTC TGC ATT GAT GTG G-MGB

*HPIV, human parainfluenza virus.

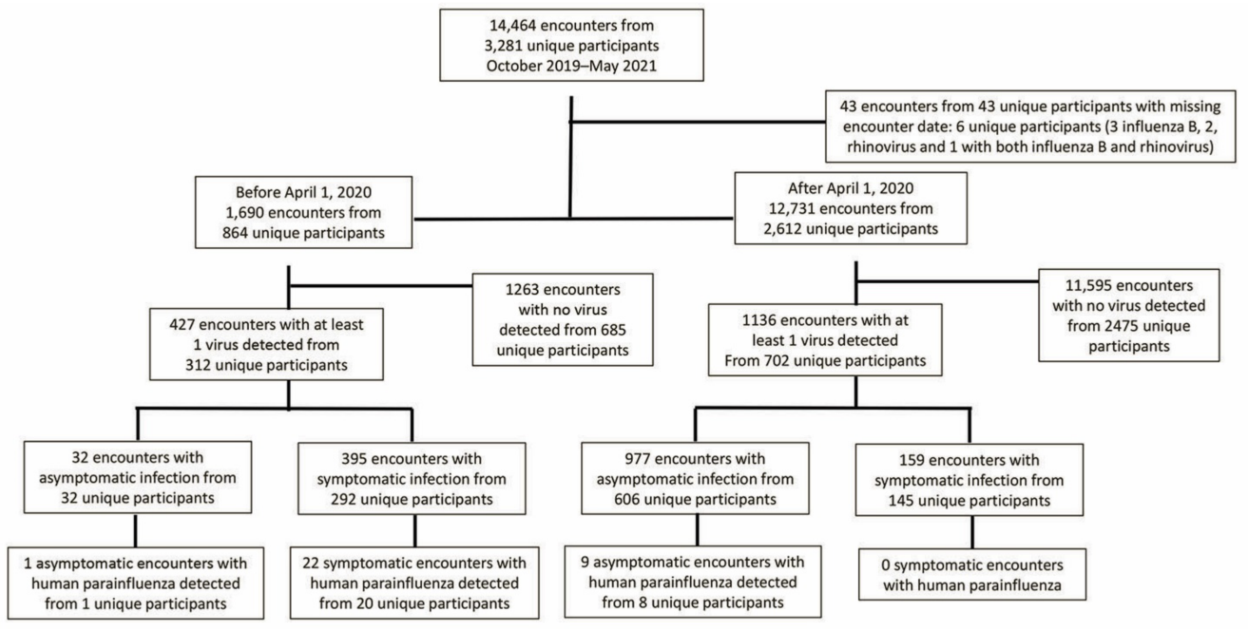

Appendix Figure 1. Homeless shelter study flowchart for human parainfluenza, King County, Washington, USA.

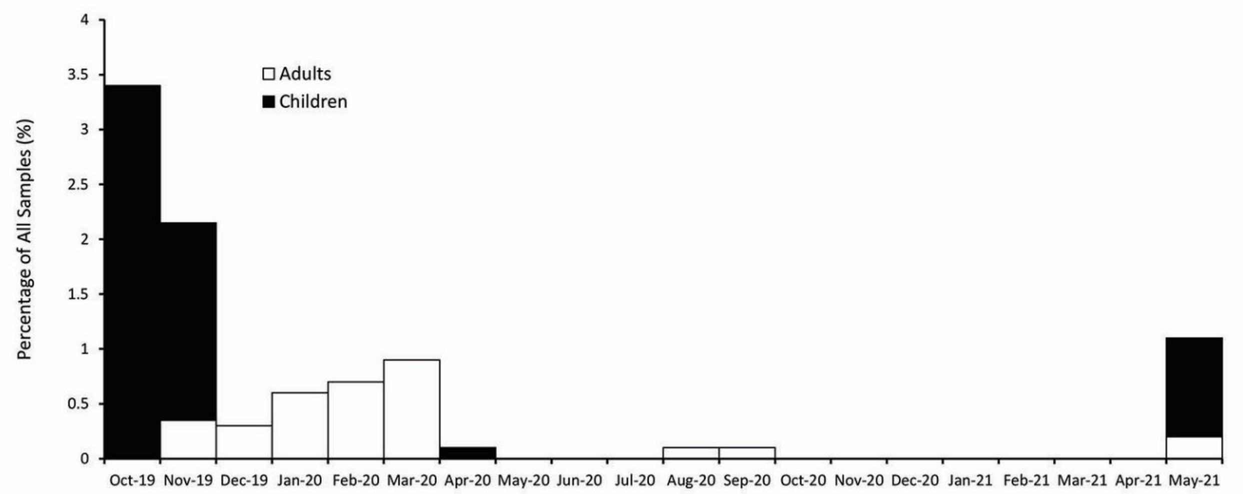

	Oct-19	Nov-19	Dec-19	Jan-20	Feb-20	Mar-20	Apr-20	May-20	Jun-20	Jul-20	Aug-20	Sep-20	Oct-20	Nov-20	Dec-20	Jan-21	Feb-21	Mar-21	Apr-21	May-21
■ Children	3.4	1.8	0	0	0	0	0.1	0	0	0	0	0	0	0	0	0	0	0	0	0.9
□ Adults	0	0.35	0.3	0.6	0.7	0.9	0	0	0	0	0.1	0.1	0	0	0	0	0	0	0	0.2

Appendix Figure 2. Homeless shelter human parainfluenza virus–positive specimens, October 2019–May 2021, King County, Washington, USA.

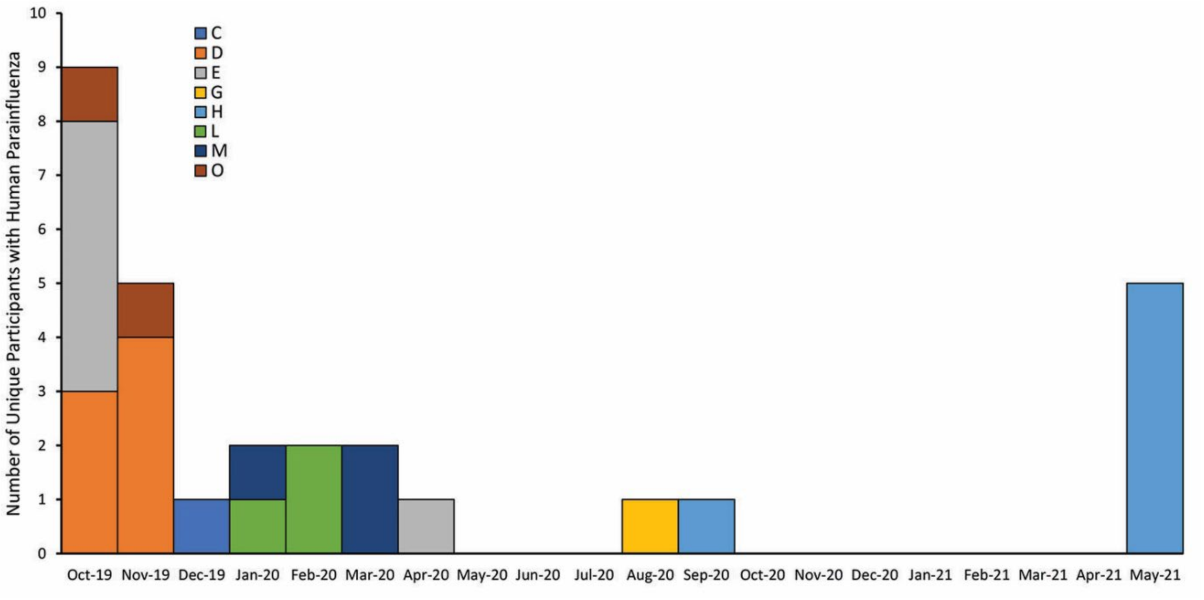

Appendix Figure 3. Unique participants with human parainfluenza virus by shelter site, King County, Washington, USA.

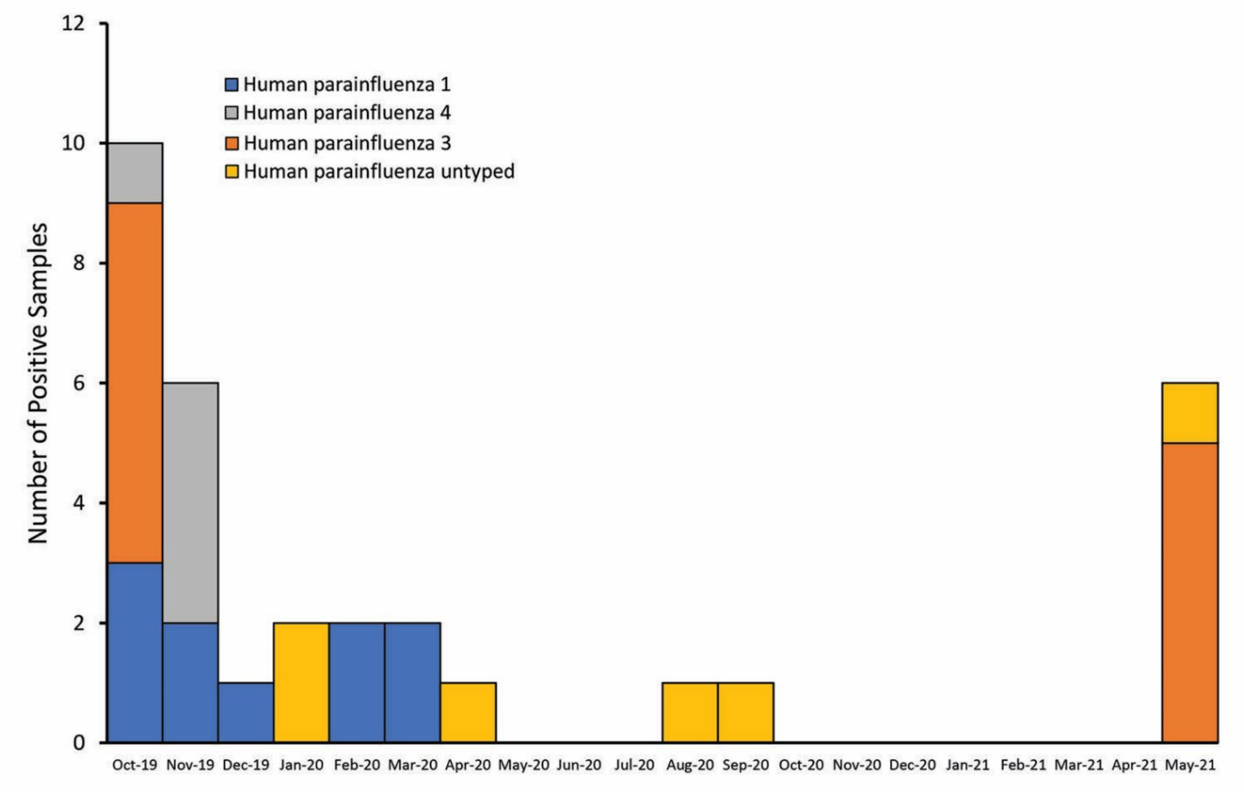

Appendix Figure 4. Human parainfluenza virus–positive specimens by human parainfluenza virus type, King County, Washington, USA.
